# Socioeconomic Inequalities in Physical Activity and Sedentary Behaviour among the Chilean Population: A Systematic Review of Observational Studies

**DOI:** 10.3390/ijerph18189722

**Published:** 2021-09-15

**Authors:** María Jesús Vega-Salas, Paola Caro, Laura Johnson, Miranda E. G. Armstrong, Angeliki Papadaki

**Affiliations:** 1 Centre for Exercise, Nutrition and Health Sciences, School for Policy Studies, University of Bristol, 8 Priory Road, Bristol BS8 1TZ, UK; laura.johnson@bristol.ac.uk (L.J.); miranda.armstrong@bristol.ac.uk (M.E.G.A.); angeliki.papadaki@bristol.ac.uk (A.P.); 2 School for Policy Studies, University of Bristol, 8 Priory Road, Bristol BS8 1TZ, UK; paola.caro@bristol.ac.uk

**Keywords:** physical activity, sedentary behaviour, socioeconomic inequalities, obesity inequalities, systematic review

## Abstract

Socioeconomic inequalities in physical (in)activity and sedentary behaviours are key mediators in obesity and health socioeconomic inequalities. Considering the high and uneven obesity rates in Chile, this review aims to systematically assess the socioeconomic inequalities in physical activity (PA) and sedentary behaviour (SB) among the Chilean population from different age groups. Peer-reviewed and grey literature were searched from inception until 31st December 2019 in PubMed, Scopus, PsycINFO, Web of Sciences and LILACS. Publications in English and Spanish, from observational studies that reported the comparison of at least one indicator of PA or SB between at least two groups of different socioeconomic positions (SEP), from the general Chilean population, were included. Data searches, screening, extraction, and quality assessment, using the Newcastle Ottawa Quality Assessment Scale for observational studies, were conducted by two independent researchers. Seventeen articles (from 16 studies) met the inclusion criteria (14 cross-sectional; two cohort). Across these, quality was considered low, medium and high for 19%, 69% and 13%, respectively. Results showed consistent evidence for a lower leisure-time PA and sitting time, and higher physical inactivity among adults from the lower, compared to the highest, SEP groups. Associations between SEP and total PA, moderate-to-vigorous PA, low PA, and transport and work-related PA were inconsistent. These findings provide insights to public health and physical activity researchers and policymakers aiming to reduce socioeconomic inequalities in PA and SB in Chile and other countries.

## 1. Introduction

Physical activity (PA) and sedentary behaviours (SB) are, respectively, protective and detrimental factors for mortality, health and non-communicable diseases [1]. Worldwide, obesity and physical inactivity reduce life expectancy by 0.7 and 2.4 years on average, respectively [2]. People from lower socioeconomic positions and those living in areas of high inequalities have a shorter life expectancy, higher mortality and worse health [3,4,5]. However, less is known about the inequalities around the obesity-related health behaviours contributing to these preventable health inequalities [6].

Chile is placed third, after Mexico and the USA, for highest adult obesity rate among the countries grouped in the Organization for Economic Cooperation and Development [7]. Nearly 25% of 5–6-year-old children and 35% of people aged >15 years in Chile are living with obesity [8,9]. Obesity-related health conditions are the top risk factor for death and disability [10] and are a burden to the Chilean economy [11], resulting in a 3.8% reduction in the country’s Gross Domestic Product per year [12].

Social inequalities affect obesity rates among the Chilean population. Data from the 2017 Chilean National Health Survey (ENS) reported an obesity prevalence among people > 15 years of 46.6% vs. 29.5% among those from lower, compared to the higher, educational groups [8]. Moreover, obesity rates were higher among women than men (38.4% vs. 30.3%, respectively) [8]. Similar trends have been reported for children aged 0–9 years (17.1% vs. 9.7%, in lower- vs. higher-income households, respectively) [13]. Obesity, at the individual level is caused by a long-term positive energy imbalance [14,15,16]. Consequently, socioeconomic–obesity inequalities suggest disparities in dietary intakes, PA and SB between different socioeconomic groups [16]. This unequal socioeconomic distribution of diet, PA and SB is related to structural inequalities at the environmental, societal and individual levels [17,18].

Several systematic reviews have suggested that higher body mass index (BMI), adiposity and/or obesity are positively associated with lower levels of PA across different age groups [19,20,21,22]. Specific types of PA, such as exercise [23] and active transport [24], have been related to lower levels of adiposity and body weight. Strong and conclusive evidence has suggested a protective effect of PA, in different dose-response, intensities and domains, on cardiovascular diseases and mortality [19,21,25,26,27]. In contrast, systematic reviews have concluded that SB is a long-term negative factor contributing to type II diabetes, cardiovascular and all-cause mortality in adults [28], and that television (TV) viewing is positively correlated with higher BMI [29]. Socioeconomic inequalities in PA have also been reported by systematic reviews including data mostly from western and high-income countries, suggesting consistently higher leisure-time PA among the higher SEP groups [30,31,32,33]. Correspondingly, for SB, higher hours of TV viewing were reported among lower educated and unemployed/retired adults, but an inverse association was reported for computer use and education [29]. Studies conducted in Chile have not been considered by the aforementioned reviews, mostly due to the geographical location (not European or North American), language barriers (not conducted in English), the changing development status (from middle-income country (MIC) to high-income country in 2013) [34], or not meeting other inclusion criteria.

The rapid rise in obesity rates in Chile and their social inequality is similar to those observed in other low-and middle-income countries (LMICs). Therefore, understanding how PA and SB relate to SEP in Chile, as an exemplar of a rapidly developing country, is of relevance to other countries. To our knowledge, no systematic review to date has investigated the socioeconomic inequalities in PA and SB in Chile, which would be important to inform policies aiming at reducing PA and SB inequalities in Chile, and other countries with similar epidemiological transitions. The aim of the current study was, therefore, to systematically assess the socioeconomic inequalities in PA and SB among the Chilean population, and, if available, conduct a comparison between SEP and PA or, SB, stratified by gender, age group or body weight status. Also, a comparison between studies conducted at different stages of the Chilean nutritional transition, which has contributed to the rising obesity rates in the country [35], will be included to assess changes in SEP inequalities in PA and SB through time.

## 2. Materials and Methods

**Figure 1:**
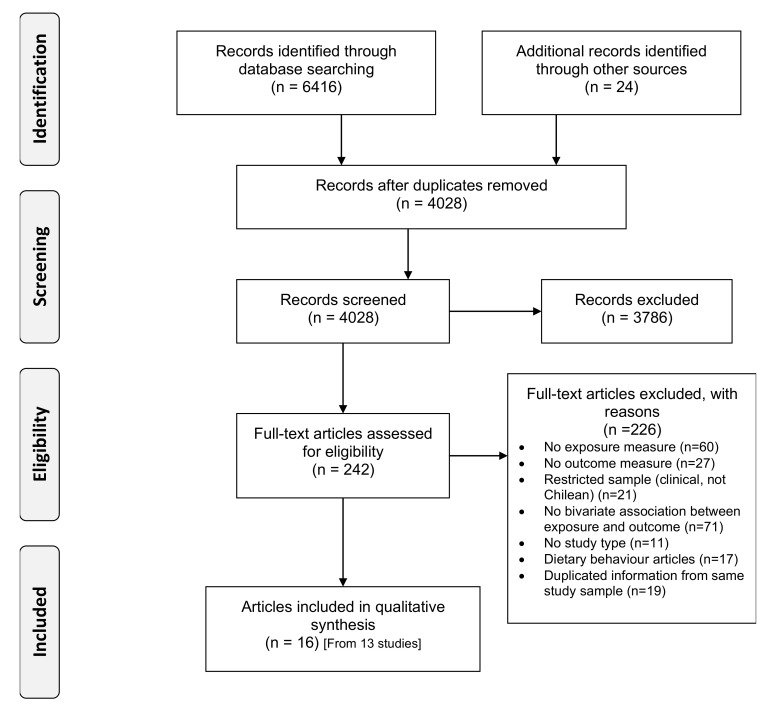
Preferred Reporting Items for Systematic reviews and Meta-Analyses (PRISMA) flow diagram of literature *Scheme*.

This systematic review was conducted following the Preferred Reporting Items for Systematic Reviews and Meta-Analyses Equity extension (PRISMA-E 2012) guidelines [36] (Figure 1 and PRISMA-E checklist Supplementary Table S1). The review protocol for a larger systematic review assessing socioeconomic inequalities in diet, PA and SB among the Chilean population was registered in PROSPERO (CRD42018096925).

### 2.1. Search Strategy

Searches were conducted in MEDLINE (via PubMed), Scopus, PsycINFO, Web of Science, and Latin American and Caribbean Health Sciences Literature (LILACS) for articles published from inception until 31 December 2019. As a secondary aim was to contrast studies conducted at different stages of the nutritional transition, searches were not date restricted from inception. Searches included peer-reviewed articles and grey literature available at OpenGray and national and international organisations’ websites (e.g., Chilean Ministries, World Health Organisation) (Supplementary Tables S2–S7). Reference lists of included articles were hand-searched and checked for potential inclusion. Searches were conducted by two researchers independently (M.J.V.-S. and P.C.). 

### 2.2. Inclusion Criteria

Articles published in English and Spanish from observational studies conducted in Chile were eligible for inclusion. Studies conducted among the general population, regardless of age, and without excluding participants based on sex, were included. Studies were excluded if conducted in clinical settings or were developed as weight-related interventions (except when baseline measures of interest were available) or focused on chronic diseases that may impact on weight (e.g., diabetes, cancer, HIV), or sample sizes included <100 participants.

Articles were included if they reported differences between two or more groups of different SEP in at least one indicator of PA, SB or dietary intake. Due to the number and heterogeneity of the different indicators extracted from the included studies, we decided to report the results of the dietary intake elsewhere [37]. Indicators of PA and SB were included if they were presented by domain (e.g., leisure-time, work and transport PA, sitting, TV viewing, computer use) and/or intensity (e.g., low, moderate, or vigorous), and/or duration of the activity (e.g., minutes, hours) or the Metabolic Equivalent (MET) or frequency of PA engagement (e.g., Frequency of leisure-time PA sessions per week) or compliance with recommendations (e.g., ≥150 min/week or ≥600 METs/week) [38,39].

### 2.3. Title Screening and Selection

Title and abstracts were screened by two independent reviewers, both fluent English and Spanish speakers (M.J.V-S and P.C.), reaching a good agreement in the pilot for the first 100 records (kappa = 0.62) [40]. Discrepancies during the pilot test were resolved with other authors (L.J. and A.P.), and an excellent inter-rater agreement (kappa = 0.93) was obtained for the remaining titles and abstracts. All discrepancies were discussed until consensus was reached. Authors were contacted if clarification on any aspect of an article was required. 

If multiple articles of the same study were considered for inclusion but reported the same PA or SB and SEP indicators, only the publication with the most complete data for the purposes of the current review was included. If multiple articles from the same study reported different indicators, each publication was individually included in the review. 

### 2.4. Data Extraction

Data were extracted from each included article in a piloted table (Supplementary Table S8). If an article reported associations of multiple SEP, PA or SB indicators, data extraction was conducted individually for each indicator. Due to the heterogeneity in indicators and measurements and the various approaches to adjustment across studies, we extracted unadjusted associations from bivariate tables between each PA or SB indicator and at least two SEP groups, aiming to reduce the impact of the variability of confounders and mediators, and thus allowing comparisons across studies.

### 2.5. Outcomes

#### 2.5.1. Physical Activity and Sedentary Behaviour Factors

Physical activity was defined as “any bodily movement produced by skeletal muscles that requires energy expenditure” [38]. Due to the diverse methods of reporting PA across the articles, data were summarised by PA domain (total, leisure-time, work and transport), intensity (low, moderate and vigorous) and/or by the lack of PA (physical inactivity) [41]. Similarly, SB was defined as “any waking behaviour characterized by an energy expenditure of ≤1.5 METs, while in a sitting, reclining or lying posture” [42]. Results were summarised by domain (sitting time and television viewing).

#### 2.5.2. Socioeconomic Position

The socioeconomic position (SEP) indicates the relative position of individuals or groups according to the differential access to the actual capital or resources and prestige status within a society [43,44]. SEP stratifies and determines health opportunities and outcomes [45,46,47]. Only individual or household level SEP indicators, based on education, occupation and/or income, or composite indices, reported directly by the participants, were eligible for inclusion. We decided to exclude area-level (e.g., borough or municipality) or institutionally based (e.g., school type attendance) SEP indicators, as this information usually reflects aggregated or administrative information not reported directly by the participants. For comparison and analysis purposes, the low SEP indicator was compared against the middle to high SEP indicator.

### 2.6. Quality Assessment and Risk of Bias

The quality of the included articles was assessed with an adaptation of the Newcastle Ottawa Quality Assessment Scale (NOQAS) (max. 10 points) for cohort and cross-sectional studies [48] and was performed by two reviewers independently (M.J.V.-S. and P.C.) (Supplementary Table S9).

### 2.7. Data Analysis

After extracting the data, we estimated the magnitude of the differences in PA and SB between the higher and lower SEP groups. Calculations included relative differences between time spent in an activity (e.g., minutes per day) or by Odds Ratios (OR) for proportions (e.g., % of participants not meeting PA guidelines). The following formulas used by previous systematic reviews assessing inequalities in health-related behaviours [16,49] were applied:

(1) $$\mathrm{Relative}\;\mathrm{difference}\left(\%\right)=\frac{\left(\mathrm{value}\;\mathrm{high}\;\mathrm{SEP}\;\mathrm{group}-\mathrm{value}\;\mathrm{low}\;\mathrm{SEP}\;\mathrm{group}\right)}{\left(\mathrm{value}\;\mathrm{high}\;\mathrm{SEP}\;\mathrm{group}\right)}\;\times \;100$$

(2) $$\mathrm{OR}=\frac{\rho \;\mathrm{high}\;\mathrm{SEP}}{\left(1-\rho \right)\;\mathrm{high}\;\mathrm{SEP}}/\frac{\rho \;\mathrm{low}\;\mathrm{SEP}}{\left(1-\rho \right)\;\mathrm{low}\;\mathrm{SEP}}$$

Following the classification used by Giskes et al. [16], associations were classified as no association (<10% relative difference or OR 0.91–1.0), moderate (10–20% relative difference or OR 0.80–0.90) and large (>20% relative difference or OR < 0.80). Results were presented in tables and synthesised in harvest plots [50], stratifying by total population and gender, and by quality score (3 groups).

## 3. Results

### 3.1. Included Articles

**Figure 2:**
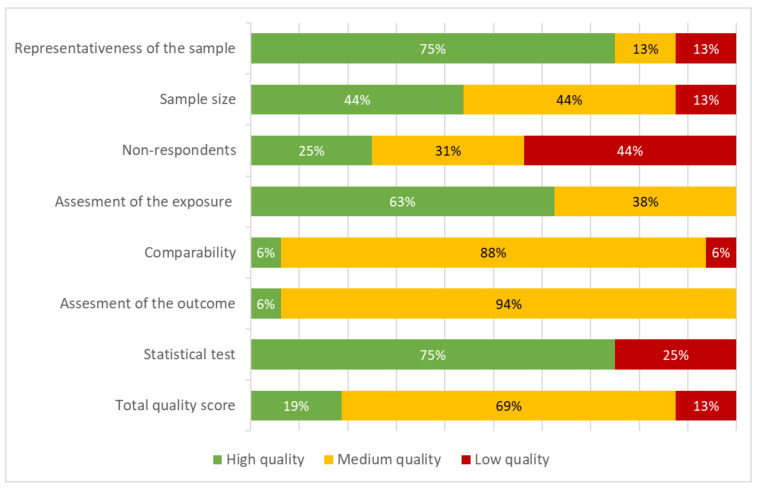
NOQAS quality assessment of included publications (N = 16).

The search and study selection processes are illustrated in Figure 1. Out of 4028 unique records, 242 full-text articles were assessed for eligibility and 16 articles (representing 13 separate studies) were included. Table 1 depicts the characteristics of the 16 articles included. Sample sizes ranged from 472 to 9503 participants, with 14 articles presenting samples sizes >1000 participants. One article collected data during the Chilean nutritional transition (1960–1989) [51], one during the 1990s [52], and all the remaining 14 articles collected data after the 2000s. The quality assessment’s summary is presented in Figure 2 and Supplementary Table S10.

### 3.2. Physical Activity

Fourteen out the fifteen articles reporting associations between PA and SEP used self-report questionnaires derived from validated international assessments such as the Global Physical Activity Questionnaire (GPAQ) (five articles) and the International Physical Activity Questionnaire (IPAQ) (three articles). The SEP assessment was conducted using education (five articles), multidimensional indexes (four articles), a combination of two or more SEP indicators (five articles) and income (one article). All fifteen articles included data on adults only. Overall, results show variable evidence of association between PA and SEP among adults.

**Figure 3:**
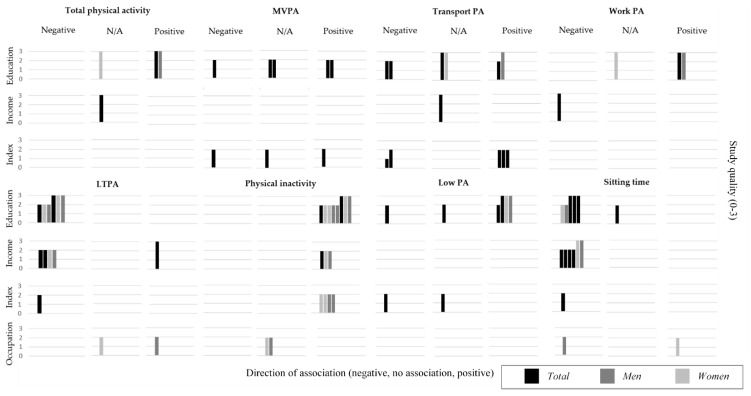
Summary of evidence for associations between socioeconomic position and physical activity and sedentary behaviour. Each row represents a dimension of socioeconomic position, and each column represent the direction of the association between socioeconomic position indicators and physical activity and sedentary behaviour measurement. Relative differences ≥10% or OR<0.80 were categorised as negative association (lower activity among lower SEP groups, compared to the higher) or positive association (higher activity among lower SEP groups, compared to the higher). Relative differences <10% were classified as no association (N/A). Each bar represents an association between SES and physical activity or sedentary behaviour. The quality assessment scores from the articles are indicated by the height of the bars (1 = Quality scores ≤4.5; 2 = Q.S.>4.5 and <7; 3 = Q.S.≥7). Studies conducted among adults are indicated with full-tone (black) bars, only adult men are presented half-tone (grey) and adult women with quarter-tone (light grey). PA: Physical Activity; MVPA: Moderate and/or vigorous PA; LTPA: Leisure-time PA.

Two articles examined a total of four associations between total PA and SEP (Supplementary Table S11 and Figure 3). A strong association was reported for higher total PA among the lower educated SEP group among the total population and men (Δ = 24.2 and 39.6%). However, the remaining two associations among income groups and women were not meaningful (<10%).

Three articles examined eight associations between moderate and/or vigorous PA (MVPA) and SEP (Supplementary Table S11 and Figure 3). Three associations reported a higher MVPA (Δ = 47.7%, OR = 2.13 and 1.53) among the lower SEP groups. In contrast, two associations suggested a lower MVPA among this same SEP group (OR = 0.52 and 0.88, respectively). The remaining three associations were not meaningful (<10%).

Seven articles reported on 12 different associations between transport-related PA and SEP among adults (Supplementary Table S12 and Figure 3). Overall, inconsistent results were reported for walking or cycling for transportation (active transport). Four associations suggested a lower active transport among the lower SEP groups, either measured by minutes per week and % of participants engaging in active transportation regularly (Δ = −26.9 and −16.3%, OR = 0.27 and 0.59, respectively). In contrast, five associations reported higher active transport among the lower SEP groups, measured either by minutes per day and as % of participants engaging in active transport (Δ = 19.9% and OR between 1.17 and 5.58, respectively). The remaining three associations reported relative differences <10%.

Two articles, based on the high-quality national survey ENS 2009–2010, reported four associations between work-related PA and SEP (Supplementary Table S12 and Figure 3). Two associations reported higher work MVPA among lower educated men and the total population, but not among women, measured either by METs or minutes/day (Δ = 78.1, 42.5 and 9.1%, respectively). In contrast, a small inverse association was reported for income groups (Δ = −10.7%).

Five articles examined 14 different associations between leisure-time PA (LTPA) and SEP (Supplementary Table S13 and Figure 3). Overall, all six associations using education as the SEP indicator presented a consistent case for lower LTPA among the lower SEP groups (measured either by time or frequency), compared to the higher (ranging between Δ = −19.6 and −68.3% and OR = 0.22 and 0.54). Similarly, four associations using income, and one using a composite index reported strong associations for lower LTPA among the lower SEP groups (OR between 0.31 and 0.87). In contrast, two associations using income and occupation presented a higher LTPA among the lower SEP groups (Δ = 20.8% and OR = 2.04, respectively). One association using occupation was not meaningful <10%.

Seven articles reported on 17 associations between physical inactivity and SEP among adults (Supplementary Table S14 and Figure 3). Overall, consistent results indicated higher physical inactivity (lower MVPA) among the lower SEP groups. All associations, except those using occupational SEP measurements, presented a strong case for higher physical inactivity according to the 2005 Chilean PA guidelines (LTPA <three times-per-week for <30 min each time) [66] (OR between 1.82 and 6.25). Lower SEP groups also reported a higher % for not engaging in any kind of leisure-time PA over the last month (OR between 1.71 to 5.30).

Four articles reported on eight associations between low PA or not meeting the WHO PA guidelines (<600 METs/week) and SEP (Supplementary Table S14 and Figure 3). Overall, some inconsistencies were reported for the % of participants not meeting the PA guidelines, with ORs ranging from 0.46 to 2.06. Four associations reported a higher % of participants from the lower SEP groups not meeting the guidelines (OR between 1.17 and 2.06), whereas two associations from the same article reported the opposite finding (OR = 0.46 and 0.68). The two remaining were non-significant associations (<10%).

### 3.3. Sedentary Behaviours

Six out of the seven articles reporting associations between SB and SEP used self-report questionnaires, mostly derived from validated international assessments such as GPAQ and IPAQ. The SEP assessment was conducted using education (two articles), multi-dimensional indexes (one article), income (one article) and a combination of two or more SEP indicators (three articles).

Five articles examined 15 associations between sitting time and SEP among adults (Supplementary Table S15 and Figure 3). Eleven associations showed a consistent lower sitting time among the lower SEP groups, either measured by minutes per day, or % of participants engaging more in sitting time (Δ between −14 and −24.4%, OR between 0.26 and 0.44). Two large national surveys reported lower chances among lower SEP groups to be identified with the statement “I spent most time seated and walk little” (OR between 0.1 to 0.49). In contrast, only one association including women from the lower occupational group showed an opposite direction (OR = 1.82).

One article among children and adolescents assessed four associations between TV viewing and SEP (Supplementary Table S15). Children aged 3–5 years from the lower SEP reported spending more time viewing TV and using TV more during weekdays, compared to their higher SEP counterparts (Δ = 14.9% and OR = 1.56, respectively). In contrast, the associations among adolescents aged 12–14 years were weaker but inverse, with adolescents from the lower SEP engaging less in television viewing (Δ = −11.1% and OR = 0.74, respectively).

## 4. Discussion

We conducted a systematic review of observational studies to examine socioeconomic differences in PA and SB among the Chilean population. To our knowledge, this is the first systematic review assessing the evidence of socioeconomic inequalities in PA and SB in Chile. We found strong and consistent evidence for lower leisure-time PA and sitting time, and higher physical inactivity, among the lower SEP groups, compared to the higher. However, inconsistent associations between SEP and total PA, MVPA, transport and work-related PA, and low PA were observed. All but one article included samples on adults only, with one article reporting on a higher SB (TV viewing) among children from the lower SEP, but an inverse association among adolescents. These observed SEP inequalities in PA and SB might be contributing to the socioeconomic gradients in obesity rates and should be taken into account when developing future interventions, strategies and policies to tackle obesity in Chile.

Our findings suggesting a consistent lower leisure-time PA among the lower SEP groups agrees with several reviews of articles among adults from Europe and Western-developed countries [30,31,33,67]. Moreover, our study reporting that adults from lower SEP engaged more in physical inactivity is a novel finding, not observed in the aforementioned reviews. There is sufficient evidence about the health benefits of LTPA and the detrimental effects of physical inactivity for body weight, cardiovascular diseases, cancer and mortality [1,25,68,69]. Our findings for physical inactivity mirror the LTPA results, as the measurement for the former combines a mixture of indicators around the lack of leisure-time MVPA. Another proxy of physical inactivity, not meeting the WHO PA guidelines (≤600 MET-min/week), showed less conclusive results. Longitudinal studies from high-income countries (not including Chile) have reported a persistent and even widening gap between LTPA and socioeconomic inequalities over time [67,70,71,72]. Our conclusive findings suggest LTPA-SEP inequalities can relate to the unequal impact of public health policies promoting LTPA across the population. As most PA guidelines suggests increasing LTPA, this advice has been taken up more by the higher SEP groups, but not among the lower SEP groups [73]. Further studies in the Chilean population should address the reasons underpinning the lower LTPA among the lower SEP groups and consider their particular barriers when planning public health policies aiming at increasing PA levels across the population.

The direction and strength of the SEP inequalities in PA differed considerably by domain, SEP indicator and gender. Some evidence of an association suggests a higher total and work-related PA among the lower educated men and total population, but not among women or when using an income-based indicator. An inconsistent direction of associations was reported for transport-related PA among the total population, but showing similar gender differences as for total and work-related PA. Conversely, LTPA and physical inactivity showed no clear gender differences and stronger associations when using education but was weaker when using occupation. The smaller relative difference in transport, work and total PA among women reflects an overall lower PA in these domains when compared to men. These findings might explain the obesity-gendered pattern in Chile, where women display higher obesity rates, compared to men [8,60,74]. 

Our findings, suggesting a lower SB (sitting time) among the lower SEP adults, contrast with the lack of consistent results reported by two reviews conducted among adults from mainly western and high-income countries [29,75]. The review by Prince et al. [75] suggests a consistent association for higher transport and work-related SB among the higher SEP groups, whereas the review conducted by Rhodes et al. [29] suggests a higher TV viewing among lower educated groups, an inverse association for computer use and no clear association with general sitting time. Our review could not identify specific domains for SB, rather overall measures of sitting time among adults and TV viewing among children. Among children, mixed results showed a lower TV viewing among adolescents from the lower SEP groups, but the opposite association among younger children. In contrast to our findings for PA, the direction and strength of the SEP inequalities for sitting time among adults does not differ considerably by SEP indicator. Furthermore, no clear gender patterns among adults were identified for sitting time.

The decrease in PA and increase in SB during the last decades, and the consequent increase in obesity rates, can relate to the nutritional transitions and modernisation processes around the globe [76]. Strong evidence suggests that the built environment can promote or discourage physical activity and sedentary behaviours [77] and is key for promoting/preventing obesogenic environments [18]. A study conducted in Santiago city concluded that lower-income areas have less access to public transport and urban quality walking environment [78] and fewer green areas [79] compared to higher-income areas. The PA built environment determinants are also gender-patterned [80], including a higher perceived insecurity around walking environments among middle-income women from Santiago city, when compared to men [81]. In Chile and Latin America, PA interventions have focused mostly on improving physical fitness at school level but have been inconclusive for body weight reduction [82,83]. However, these interventions do not report time-based or frequency changes for physical activity domains [84,85], and therefore were not included in our review. Insufficient but promising evidence for PA interventions reducing body weight at the population level include individually adapted health behaviour change and changes to the built environment [86]. Moreover, community-based interventions among socio-economically disadvantaged populations have shown greater effectiveness when delivered at group level (e.g., family or household) than individual level [87]. Interventions aiming to increase PA and reduce SB among the more deprived populations must consider the individual, social, socioeconomic, and environmental difficulties these groups face in their everyday life. Together with improving access and distribution of healthy diets among the lower SEP populations, policies aimed at reducing obesity rates should incorporate a gender and equity-based component to avoid widening social inequalities in obesity-related behaviours [88,89]. Due to the lowest overall PA among the lower SEP groups, built environment interventions targeting the more deprived areas can have even greater impacts in improving PA and overall health [90]. 

### Strengths and Limitations

This is the first study, to our knowledge, to systematically examine the evidence for socioeconomic inequalities in PA and SB in Chile. Inclusion of peer-reviewed and grey literature in English and Spanish and conducting the review under the PRISMA-E guidelines [36] strengthens our study and minimises the risk of publication bias [91]. Our evidence synthesis in harvest plots, including the NOQAS quality assessment scores, is a novel and comprehensive method for presenting results. However, our study has some limitations. The small number of studies and the diverse cut-off points and large heterogeneity of PA, SB and SEP indicators may have limited our ability to draw robust conclusions or to provide a quantitative pooled analysis. To diminish the variation of the size of associations and allow similar comparisons across studies, we decided to extract unadjusted associations between PA or SB indicators and two SEP groups. However, unadjusted associations are constrained for residual confounding and mediation associations. We therefore decided to use a conservative cut-off point (10%) for assessing the magnitude and relevance of the associations in our evidence synthesis. All but two articles were cross-sectional in design, and therefore it is not possible to conclude if belonging to a lower SEP group will cause a lower PA/SB engagement or vice versa (reverse causality). Despite these limitations, our review provides evidence on all available PA and SB domains and SEP indicators included in studies in the Chilean population to 31 December 2019. Future studies should aim to use similar cut-off points, definitions and measurements for PA, SB and SEP indicators, offering fairer comparisons between studies. Studies should also aim to report multiple SEP indicators, as PA, SB and health-related behaviours can vary according to the SEP indicator used [30,31,44]. Further qualitative and longitudinal studies should explore how the PA and SB social inequalities are expressed among the population across the life course and explore in depth how these inequalities contribute to widening the social gradients in obesity rates. Moreover, studies capturing the potential bidirectional associations between PA, SB and obesity and the role of SEP inequalities are needed.

All except one article in our review [56] used self-reported questionnaires to assess PA and SB, and all indicators measuring sitting time were assessed using a single self-report question. These methodological limitations may introduce a reliability issue on our estimates. Self-report measures over- and underestimate PA levels, and underestimate SB time, when compared to direct measures (e.g., accelerometers) [92,93]. Further studies should aim to include objective measurements for PA and SB or assess reliability from self-report questionnaires when it is not possible to measure these behaviours with a more objective assessment method [94]. Studies classified as being of low quality did not report non-response characteristics, had lower response rates, and did not include key inferential statistics for assessing the degree of strength of evidence of associations (standard deviation or confidence intervals, and *p*-values). Future studies should aim to conduct rigorous and high-quality research and transparently provide key information for assessing the quality of the studies conducted. Some final limitations were the lack of studies conducted during the nutritional transition (1960–1989), limiting our ability to draw comparisons between studies conducted during and after this period, and the lack of studies assessing SEP differences in PA among children. Future research is needed to assess PA engagement by domain, intensity and frequency among children.

## 5. Conclusions

This review is the first, to our knowledge, to provide a comprehensive synthesis of the evidence on the socioeconomic inequalities in PA and SB in the Chilean population. Overall, we found consistent evidence for a lower leisure-time PA and sitting time, and higher physical inactivity among adults from the lower SEP groups, and inconsistent associations between SEP and total PA, MVPA, low PA, and transport-and-work-related PA. Furthermore, our findings suggesting a gendered pattern for work, transport and total PA are a novel finding, showing the relevance of studying further the intersections between gender and SEP in PA and health studies. Our review provides a complete picture of the SEP inequalities in two key health behaviours, which, for policy development, should be viewed alongside inequalities in dietary intakes [37]. This also contributes evidence for future research to encourage the implementation of comprehensive and broader policies aiming at reducing obesity and wider health inequalities by promoting PA and reducing sedentary time.

## Figures and Tables

**Table 1:** Study characteristics.

Author	Study Name/ Year Data Collection	Location	Study Design	Sample Population	Sample Size	Response Rate	Age Group	SEP Indicator	PA/SB Assessment Method	Quality Score
Dillman Carpentier et al., (2019) [53]	FEChiC and GOCS 2016	Santiago	L	Children	N = 879 CH/ N = 753 ADOL	N/R	3–5 y and 12–14 y	Mother’s education level: 3 groups (less than high school or lower vs. more than high school)	Self-report	5.5
Aguilar-Farias et al., (2019) [54]	NES 2014 and 2015	Chile	C	Adults	N = 5057 (2014) N = 5664 (2015)	N/R	≥18 y	Household index (AIM): 3 groups (low vs. high)	Self-report	4.5
Barranco-Ruiz et al., (2019) [55]	2015	Valparaíso	C	Adults	N = 496	N/R	≥18 y	Household index (AMAI): 3 groups (low vs. high)	Self-report (IPAQ)	3
Berrios et al., (1990) [51]	1986–1987	Santiago	C	Adults	N = 1203	87%	≥15 y	Household index (Graffar’s modified scale): 3 groups (low vs. high + medium-high)	Self-report	6
Celis-Morales et al., (2011) [56]	GENADIO 2008	Santiago, Los Rios, Bio-Bio	L	Adults	N = 472	54%	20–60 y	Household index (ESOMAR): 3 groups (low vs. high) Education level: 3 groups (less than high school or lower vs. more than high school)	7-d Accelerometer	5
deMoraes Ferrari et al., (2019) [57]	ELANS 2014–2015	Chile	C	Adults	N = 9218	N/R	15–65 y	Household index: 3 groups (low vs. high) Education level: 3 groups (basic or lower vs. university degree)	Self-report (IPAQ)	6
Ministerio de Salud de Chile, (2003) [58]	ENS 2003	Chile	C	Adults	N = 3619	90%	≥18 y	Education: 3 groups (<8 years vs. >12 years)	Self-report	6
Ministerio de Salud de Chile, (2006) [59]	ENCAVI 2006	Chile	C	Adults	N = 6210	98%	≥15 y	Household income quintile: 5 groups (1st quintile vs. 5th quintile)	Self-report	6
Ministerio de Salud de Chile, (2011) [60]	ENS 2009–2010	Chile	C	Adults	N = 5434	85%	≥15 y	Education: 3 groups (<8 years vs. >12 years)	Self-report (GPAQ)	9
Celis-Morales et al., (2016) [61]	ENS 2009–2010	Chile	C	Adults	N = 5155	85%	≥15 y	Educational level: 3 groups (Primary vs. Beyond secondary) Income level: 4 groups (Lowest vs. Highest)	Self-report (GPAQ)	8
Diaz-Martínez et al., (2018) [62]	ENS 2009–2010	Chile	C	Adults	N = 4457	85%	≥15 y	Education: 3 groups (<8 years vs. >12 years)	Self-report (GPAQ)	8
Waddell et al., (2019) [63]	ENS 2009–2010	Chile	C	Adults	N = 5277	85%	≥18 y	Education: 3 groups (<8 years vs. >12 years)	Self-report (GPAQ)	6
Ministerio de Salud de Chile, (2012) [64]	ENETS 2009–2010	Chile	C	Adults	N = 9503	74%	≥15 y	Education: 7 groups (Incomplete primary vs. complete university) Household income level: 6 groups (<$136.000 vs. >$851.000 CLP) Employment status: 2 groups (non-occupied vs. occupied) Employment situation: 6 groups (dependent worker vs. owner)	Self-report	6
Ministerio de Salud de Chile, (2018) [8]	ENS 2016–2017	Chile	C	Adults	N = 6233	90%	≥15 y	Education: 3 groups (<8 years vs. >12 years)	Self-report (GPAQ)	6
Jadue et al., (1999) [52]	CARMEN 1996–1997	Valparaíso	C	Adults	N = 3120	62%	25–64 y	Education: 5 groups (no schooling vs. university)	Self-report (Baecke)	6
Serón et al., (2010) [65]	N/R	Temuco	C	Adults	N = 1535	127%	35–70 y	Household index (ESOMAR): 3 groups (low vs. high)	Self-report (IPAQ)	6.5

ADOL: adolescents; AIM: Chilean Marketing Research Association; AMAI: Mexican Association of Marketing Research and Public Opinion Agencies; C: Cross-sectional; CH: children; CLP: Chilean pesos; ELANS: Latin American Study of Nutrition and Health; ENCAVI: Chilean National Quality of Life and Health Survey; ENETS: Chilean Workers Employment Conditions, Work, Health and Quality of Life Survey; ENS: Chilean National Health Survey; ESOMAR: World Association of Market Research; FEChiC: Food Environment Chilean Cohort; GENADIO: Gens, Environment, Diabetes and Obesity; GOCS: Growth and Obesity Cohort Study; GPAQ: Global Physical Activity Questionnaire; IPAQ: International Physical Activity Questionnaire; L: Longitudinal; N/R: Not reported; NES: National Environmental Surveys; SEP: Socioeconomic position; SEP indicator (): lower and higher SEP group compared; y: years.

## Data Availability

The data presented in this study are available in Supplementary material.

## Supplementary Materials

The following are available online at https://www.mdpi.com/article/10.3390/ijerph18189722/s1, Table S1. PRISMA-E Checklist, Table S2–S7. Search terms and strategies. Table S8. Data extraction fields, Table S9. Newcastle Ottawa Quality Assessment Scale checklist, Table S10. NOQAS quality assessment per component of each included publications, Tables S11–S15. Summary of relative differences and/or odds ratios from articles assessing associations between physical activity and sedentary behaviours and socioeconomic position indicators.
