# Supplementary material for: Socioeconomic Inequalities in Physical Activity and Sedentary Behaviour among the Chilean Population: A Systematic Review of Observational Studies
Source: Int J Environ Res Public Health. 2021 Sep 15;18(18):9722. doi: 10.3390/ijerph18189722 (PMC8468594; doi:10.3390/ijerph18189722)
Supplement: Supplementary file 1 [file ijerph-18-09722-s001.zip › ijerph-1347947-supplementary.pdf]

SUPPLEMENTARY MATERIAL

Table S1. Checklist of Items for Reporting Equity-Focused Systematic Reviews.

Section	Item	Standard PRISMA Item	Extension for Equity-Focused Reviews	Pg #
**Title**				
**Title**	1	Identify the report as a systematic review, meta-analysis, or both.	Identify equity as a focus of the review, if relevant, using the term equity	1
**Abstract**				
**Structured summary**	2	Provide a structured summary including, as applicable: background; objectives; data sources; study eligibility criteria, participants, and interventions; study appraisal and synthesis methods; results; limitations; conclusions and implications of key findings; systematic review registration number.	State research question(s) related to health equity.	1
	2A		Present results of health equity analyses (e.g. subgroup analyses or meta-regression).	1
	2B		Describe extent and limits of applicability to disadvantaged populations of interest.	1
**Introduction**				
**Rationale**	3	Describe the rationale for the review in the context of what is already known.	Describe assumptions about mechanism(s) by which the intervention is assumed to have an impact on health equity.	1-2
	3A		Provide the logic model/analytical framework, if done, to show the pathways through which the intervention is assumed to affect health equity and how it was developed.	1-2
**Objectives**	4	Provide an explicit statement of questions being addressed with reference to participants, interventions, comparisons, outcomes, and study design (PICOS).	Describe how disadvantage was defined if used as criterion in the review (e.g. for selecting studies, conducting analyses or judging applicability).	2
	4A		State the research questions being addressed with reference to health equity	2
**Methods**				
**Protocol and registration**	5	Indicate if a review protocol exists, if and where it can be accessed (e.g., Web address), and, if available, provide registration information including registration number.		2
**Eligibility criteria**	6	6. Specify study characteristics (e.g., PICOS, length of follow-up) and report characteristics (e.g., years considered, language, publication status) used as criteria for eligibility, giving rationale.	Describe the rationale for including particular study designs related to equity research questions.	2-3

	6A		Describe the rationale for including the outcomes - e.g. how these are relevant to reducing inequity.	
**Information sources**	7	Describe all information sources (e.g., databases with dates of coverage, contact with study authors to identify additional studies) in the search and date last searched.	Describe information sources (e.g. health, non-health, and grey literature sources) that were searched that are of specific relevance to address the equity questions of the review.	2-3
**Search**	8	Present full electronic search strategy for at least one database, including any limits used, such that it could be repeated.	Describe the broad search strategy and terms used to address equity questions of the review.	2-3<br>Supplementary Tables 2-7
**Study selection**	9	State the process for selecting studies (i.e., screening, eligibility, included in systematic review, and, if applicable, included in the meta-analysis).		3
**Data collection process**	10	Describe method of data extraction from reports (e.g., piloted forms, independently, in duplicate) and any processes for obtaining and confirming data from investigators.		3
**Data items**	11	List and define all variables for which data were sought (e.g., PICOS, funding sources) and any assumptions and simplifications made.	List and define data items related to equity,where such data were sought (e.g. using PROGRESS-Plus or other criteria, context).	3-4
**Risk of bias in individual studies**	12	Describe methods used for assessing risk of bias of individual studies (including specification of whether this was done at the study or outcome level), and how this information is to be used in any data synthesis.		4
**Summary measures**	13	State the principal summary measures (e.g., risk ratio, difference in means).		4
**Synthesis of results**	14	Describe the methods of handling data and combining results of studies, if done, including measures of consistency (e.g., I^2) for each meta-analysis.	Describe methods of synthesizing findings on health inequities (e.g. presenting both relative and absolute differences between groups).	4
**Risk of bias across studies**	15	15. Specify any assessment of risk of bias that may affect the cumulative evidence (e.g., publication bias, selective reporting within studies).		4
**Additional analyses**	16	Describe methods of additional analyses (e.g., sensitivity or subgroup analyses, meta-regression), if done, indicating which were pre-specified.	Describe methods of additional synthesis approaches related to equity questions, if done, indicating which were pre-specified	4
**Results**				
**Study selection**	17	Give numbers of studies screened, assessed for eligibility, and included in the review, with reasons for exclusions at each stage, ideally with a flow diagram.		4 and Figure 1
**Study characteristics**	18	For each study, present characteristics for which data were extracted (e.g., study size, PICOS, follow-up period) and provide the citations.	Present the population characteristics that relate to the equity questions across the relevant PROGRESS-Plus or other factors of interest.	Table 1
**Risk of bias within studies**	19	Present data on risk of bias of each study and, if available, any outcome level assessment (see item 12).		Table 1

**Results of individual studies**	20	For all outcomes considered (benefits or harms), present, for each study: (a) simple summary data for each intervention group (b) effect estimates and confidence intervals, ideally with a forest plot.		4-10
**Synthesis of results**	21	Present results of each meta-analysis done, including confidence intervals and measures of consistency.	Present the results of synthesizing findings on inequities (see 14).	Figure 3 Supplementary Tables 11-15
**Risk of bias across studies**	22	Present results of any assessment of risk of bias across studies (see Item 15).		Figure 2
**Additional analysis**	23	Give results of additional analyses, if done (e.g., sensitivity or subgroup analyses, meta-regression [see Item 16]).	Give the results of additional synthesis approaches related to equity objectives, if done, (see 16).	Figure 3
**Discussion**				
**Summary of evidence**	24	Summarize the main findings including the strength of evidence for each main outcome; consider their relevance to key groups (e.g., healthcare providers, users, and policy makers).		10-12
**Limitations**	25	Discuss limitations at study and outcome level (e.g., risk of bias), and at review-level (e.g., incomplete retrieval of identified research, reporting bias).		12
**Conclusions**	26	Provide a general interpretation of the results in the context of other evidence, and implications for future research.	Present extent and limits of applicability to disadvantaged populations of interest and describe the evidence and logic underlying those judgments.	13
	26A		Provide implications for research, practice or policy related to equity where relevant (e.g. types of research needed to address unanswered questions).	13
**Funding**				
**Funding**	27	Describe sources of funding for the systematic review and other support (e.g., supply of data); role of funders for the systematic review.		13

From: Source: Welch V, Petticrew M, Tugwell P, Moher D, O'Neill J, Waters E, White H, and the PRISMA-Equity Bellagio Group. (2012) PRISMA-Equity 2012 Extension: Reporting Guidelines for Systematic Reviews with a Focus on Health Equity. PLoS Med 9(10): e1001333. doi:10.1371/journal.pmed.1001333

For more information: http://equity.cochrane.org/equity-extension-prisma.

Table S2. Search terms and strategy – PubMed.

	**Terms**
Exposure 1 **Physical Activity**	("Physical activity" [tiab]) OR (Exercis* [tiab]) OR (Commut* [tiab]) OR ("activ* fisic*" [tiab] OR traslad* [tiab] OR ejercicio*[tiab]) OR (Physical activity [MeSH Terms]) OR (Exercise [MeSH Terms]) OR (Commuting [MeSH Terms])
Exposure 2 **Diet**	(Nutrition* [tiab]) OR (Food* [tiab]) OR (Diet* [tiab]) OR (Eat*[tiab]) OR (Feed*[tiab]) OR "dietary intake*" [tiab] OR "energ* intake*" [tiab] OR "caloric intake*" [tiab] OR "nutriti* intake*" [tiab] OR "nutriti* assessment*" [tiab] OR "nutriti* survey*" [tiab] OR "nutriti* index*" [tiab] OR "nutriti* indices" [tiab] OR "nutriti* value*" [tiab] OR "nutriti* qualit*" [tiab] OR "dietary pattern*" [tiab] OR "dietary habit*") OR (alimento* [tiab] OR comer* [tiab] OR comida* [tiab] OR ingesta* [tiab] OR "habit* aliment*"[tiab]) OR ("Diet, Food, and Nutrition" [MeSH Terms]) OR ("Nutritional Sciences" [MeSH Terms]) OR ("Food" [MeSH Terms]) OR ("Diet" [MeSH Terms]) OR ("Eating" [MeSH Terms]) OR ("Feeding" [MeSH Terms])
Exposure 3 **Sedentarism**	((Sedentar* [tiab]) OR (("television watch*"[tiab]) OR ("TV watch*" [tiab]) OR ("screen watch*" [tiab]) OR ("computer" [tiab]) OR ("video* game*" [tiab])) OR (("sitting time"[tiab]) OR ("resting time" [tiab])) OR ("physical inactivity" [tiab]) OR (("ver television" OR "viendo TV" OR "frente a la pantalla*" OR "frente al computador*" OR "video* juego*" OR "sentad*" OR "descans*" OR "inactividad* fisica*" OR (Sedentary Lifestyle [MeSH Terms]))
Outcome **Socioeconomic factors**	(Socioeconom* [tiab]) OR ("socioeconomic factors" [tiab]) OR ("socioeconomic status" [tiab]) OR ("socioeconomic position" [tiab]) OR ("social class*" [tiab]) OR (education* [tiab]) OR (occupation* [tiab]) OR (income* [tiab]) OR (employment* [tiab]) OR (poverty [tiab]) OR (poor* [tiab]) OR (deprivation* [tiab]) OR (deprived [tiab]) OR ("factor* socioeconomic*" OR "posicion* socioeconomic*" OR "clase* social*" OR educacion* OR ocupacion* OR ingreso* OR trabajo* OR empleo* OR pobre* OR deprivacion* OR deprivado* OR ("Socioeconomic factors" [MeSH Terms]) OR ("Educational status" [MeSH Terms]) OR ("Occupations" [MeSH Terms]) OR ("Income" [MeSH Terms]) OR ("Employment" [MeSH Terms]) OR ("Poverty" [MeSH Terms])
Population **Chilean**	((Chile* [tiab] OR Chilean [tiab] OR Latinamerica* [tiab] OR "latin* americ*" [tiab] OR "South America" [tiab] OR "Southern Cone" [tiab] OR "America* del Sur" [tiab] OR "Sud America" [tiab] OR "Cono Sur" [tiab] OR Developing [tiab) AND Chile) OR (Chile [MeSH Terms])
Search Strategy	[Exposure 1 OR Exposure 2 OR Exposure 3] AND Outcome AND Population

Table S3. Search terms – Scopus.

Terms
(TITLE-ABS-KEY ("Physical* activ*" OR commut* OR exercis*)) OR (TITLE-ABS-KEY ("activ* fisic*" OR traslad* OR ejercicio*))
(TITLE-ABS-KEY (nutrition* OR food* OR diet* OR eat* OR feed* OR "dietary intake*" OR "energ* intake*" OR "caloric intake*" OR "nutriti* intake*" OR "nutriti* assessment*" OR "nutriti* survey*" OR "nutriti* index*" OR "nutriti* indices" OR "nutriti* value*" OR "nutriti* qualit*" OR "dietary pattern*" OR "dietary habit*")) OR (TITLE-ABS-KEY (alimento* OR comer* OR comida* OR ingesta* OR "habit* aliment*"))
(TITLE-ABS-KEY (sedentar* OR "television watch*" OR "TV watch*" OR "screen watch*" OR "computer" OR "video* game*" OR "sitting time" OR "resting time" OR "physical inactivity")) OR (TITLE-ABS-KEY ("ver W/3 television" OR "viendo W/3 TV" OR "frente W/3 pantalla*" OR "frente W/3 computador*" OR "video* juego*" OR "sentado*" OR "descans*" OR "inactividad* fisica*"))
(TITLE-ABS-KEY (socioeconom* OR "socioeconomic factors" OR "socioeconomic status" OR "socioeconomic position" OR "social class*" OR education* OR occupation* OR income* OR employment* OR poverty OR poor* OR deprivation* OR deprived)) OR (TITLE-ABS-KEY ("factor* W/2 socioeconomic*" OR "posicion* W/2 socioeconomic*" OR "clase* social*" OR educacion* OR ocupacion* OR ingreso* OR trabajo* OR empleo* OR pobre* OR deprivacion* OR deprivado*))
(TITLE-ABS-KEY (chile* OR chilean OR latinamerica* OR "latin* americ*" OR latinoamerica OR "South America" OR “Southern Cone” OR "America* del Sur" OR "Sud America" OR “Cono Sur” OR developing)) AND (ALL (chile))

Table S4. Search terms - Web of Science.

Terms
TS= ("Physical* activ*" OR commut* OR exercis*) OR TS= ("activ* fisic*" OR traslad* OR ejercicio*)
TS= (nutrition* OR food* OR diet* OR eat* OR feed* OR "dietary intake*" OR "energ* intake*" OR "caloric intake*" OR "nutriti* intake*" OR "nutriti* assessment*" OR "nutriti* survey*" OR "nutriti* index*" OR "nutriti* indices" OR "nutriti* value*" OR "nutriti* qualit*" OR "dietary pattern*" OR "dietary habit*")
TS= (sedentar* OR "television watch*” OR "TV watch*” OR "screen watch*" OR "computer" OR "video* game*" OR "sitting time" OR "resting time" OR "physical inactivity") OR TS= ("ver NEAR/3 television" OR "viendo NEAR /3 TV" OR "frente NEAR /3 pantalla*" OR "frente NEAR/3 computador*" OR "video* juego*" OR "sentado*" OR "descans*" OR "inactividad* fisica*")
TS= (socioeconom* OR "socioeconomic factors" OR "socioeconomic status" OR "socioeconomic position" OR "social class*" OR education* OR occupation* OR income* OR employment* OR poverty OR poor* OR deprivation* OR deprived) OR TS= ("factor* NEAR/2 socioeconomic*" OR "posicion* NEAR /2 socioeconomic*" OR "clase* social*" OR educacion* OR ocupacion* OR ingreso* OR trabajo* OR empleo* OR pobre* OR deprivacion* OR deprivado*)
TS= (Chile* OR chilean OR latinamerica* OR "latin* America*" OR "South America" OR "America* del Sur" OR "Sud America" OR “Cono Sur” OR “Southern Cone” OR developing) AND (CU= (Chile) OR TS=(Chile)))

Table S5. Search terms - PsyINFO (via Ovid).

Terms
(physical activity or "Exercis*" or "Commut*" or "activ* fisic*" or "traslad*" or "ejercicio*").ab,ti. or (Physical activity or Exercise or Commuting).mh,sh.
("Nutrition*" or "Food*" or "Diet*" or "Eat*" or "Feed*" or "dietary intake*" or "energ* intake*" or "caloric intake*" or "nutriti* intake*" or "nutriti* assessment*" or "nutriti* survey*" or "nutriti* index*" or "nutriti* indices" or "nutriti* value*" or "nutriti* qualit*" or "dietary pattern*" or "dietary habit*" or ("alimento*" or "comer*" or "comida*" or "ingesta*" or "habit* aliment*")).ab,ti. or (Diet or Nutrition or Food or "Eating Behavior" or "Food Intake" or "Eating Attitudes").mh,sh.
("Sedentar*" or "television watch*" or "TV watch*" or "screen watch*" or "computer" or "video* game*" or "sitting time" or "resting time" or "physical inactivity" or ("ver ADJ3 television" or "viendo ADJ3 TV" or "frente ADJ3 pantalla*" or "frente ADJ3 computador*" or "video* juego*" or "sentad*" or "descans*" or "inactividad* fisica*")).ab,ti. or Sedentary behaviour.mh,sh.
("Socioeconom*" or "socioeconomic factors" or "socioeconomic status" or "socioeconomic position" or "social class*" or "education*" or "occupation*" or "income*" or "employment*" or poverty or "poor*" or "deprivation*" or deprived or "factor* ADJ2 socioeconomic*" or "posicion* ADJ2 socioeconomic*" or "clase* social*" or "educacion*" or "ocupacion*" or "ingreso*" or "trabajo*" or "empleo*" or "pobre*" or "deprivacion*" or "deprivado*").ab,ti. or (Socioeconomic level or socioeconomic class or socioeconomic status or Educational Attainment Level or Occupations or Income level or Employment status or Poverty).mh,sh.
("Chile*" or "Chilean" or "Latinamerica*" or "latin* americ*" or "South America" or "Southern Cone" or "America* del Sur" or "Sud America" or "Cono Sur" or Developing).ab,ti. and chile.af.

Table S6. Search terms - LILACS (via BVS).

Terms
(tw:(“Physical activity” OR commut$ OR exercis$ OR "actividad fisica" OR traslad$ OR ejercicio$))
(tw:(Nutrition$ OR Food$ OR Diet$ OR Eat$ OR Feed$ OR "dietary intake" OR "energy intake" OR "caloric intake" OR "nutritional intake" OR "nutritional assessment" OR " nutritional survey" OR " nutritional index" OR "nutritional indices" OR "nutritional value" OR "nutritional quality" OR "dietary pattern" OR "dietary habit" OR alimento$ OR comer$ OR comida$ OR ingesta$ OR "habito alimentario" OR "habito alimenticio"))
(tw:(Sedentar$ OR "television watch" OR "TV watch" OR "screen watch" OR computer$ OR "video game" OR "sitting time" OR "resting time" OR "physical inactivity" OR "ver television" OR "viendo TV" OR "frente pantalla" OR "frente computador" OR "video juego" OR sentad$ OR descans$ OR "inactividad fisica"))
(tw:(Socioeconom$ OR "socioeconomic factors" OR "socioeconomic status" OR "socioeconomic position" OR "social class" OR education$ OR occupation$ OR income$ OR employment$ OR poverty OR poor$ OR deprivation$ OR deprived OR "factor socioeconomico" OR "posicion socioeconomica" OR "clase social" OR educacion$ OR ocupacion$ OR ingreso$ OR trabajo$ OR empleo$ OR pobre$ OR deprivacion$ OR deprivado$))
tw:(Chile$ OR Chilean OR Latinamerica$ OR "latin america" OR "South America" OR "Southern Cone" OR "America del Sur" OR "Sud America" OR "Cono Sur" OR Developing) AND ((cp:(chile)) OR (pais_afiliacao:(chile)) OR (pais_assunto:(chile)))

Table S7. Search terms and strategy – Grey literature.

	**Terms**
Exposure 1 **Physical Activity**	("Physical activity" OR Exercise OR Commuting OR "actividad fisica" OR OR ejercicio)
Exposure 2 **Diet**	(Nutrition OR Food OR Diet OR Eat OR Feeding OR "dietary intake" OR "energy intake" OR "caloric intake" OR "dietary pattern" OR "dietary habit") OR (alimentación OR comer OR comida OR ingesta OR "hábito alimentario"
Exposure 3 **Sedentarism**	(Sedentary OR "television watching OR "TV watching" OR "screen watching" OR "computer" OR "video game" OR "sitting time" OR "resting time" OR "physical inactivity" OR "ver television" OR "viendo TV" OR "frente a la pantalla" OR "frente al computador" OR "video juego" OR "sentado" OR "descanso" OR "inactividad fisica"
Outcome **Socioeconomic factors**	(Socioeconomic OR "socioeconomic factors" OR "socioeconomic status" OR "socioeconomic position" OR "social class" OR education OR occupation OR income OR employment OR poverty OR poor OR deprivation OR deprived OR "factor socioeconomico" OR "posición socioeconomica" OR "clase social" OR educacion OR ocupacion OR ingreso OR trabajo OR empleo OR pobre OR deprivacion OR deprivado)
Population **Chilean**	Chile OR Chilean OR Latinamerica OR "latin america" OR "South America" OR "Southern Cone" OR "America del Sur" OR "Sud America" OR "Cono Sur" OR Developing
General search strategy	(diet OR physical activity OR sedentarism AND socioeconomic position AND Chile)
Specific search Strategy	[Exposure 1 OR Exposure 2 OR Exposure 3] AND Outcome AND Population

Table S8. Data extraction fields

- Author
- Journal
- Year of publication
- Location of study (country/region/city)
- Study design
- Study/survey name
- Date of data collection
- Sample population (children/adolescents/adults)
- Sampling recruitment techniques
- Sample size
- Study response rate
- Population and participant characteristics
- Sample’s age
- SEP indicator
- SEP assessment method
- Physical activity factors
- Physical activity assessment method
- Direction and magnitude of the association between SEP and physical activity
- Sedentary behaviour factors
- Sedentary behaviour assessment method
- Direction and magnitude of the association between SEP and sedentary behaviours

Table S9. Newcastle - Ottawa Quality Assessment Scale (adapted for cross sectional studies) (10 points max.)

Selection: (Maximum 5 stars)

1) Representativeness of the sample:
   a) Truly representative of the average in the target population* (1 star)
      - Clear sample frame
      - Multistage sampling
      - Random sampling in all sampling stages
   b) Somewhat representative of the average in the target population* (0.5 star)
      - Single-stage sampling
      - Non-random sampling on at least 1 sampling stages (e.g. random of students with a school but non-random sampling of schools)
   c) Selected group of participants (0 star)
      - Convenience sampling or selection based on a particular characteristic of individuals
   d) No description of the sampling strategy (0 star)
2) Sample size:
   a) Justified and satisfactory * (1 star)
      - Sample size justified and estimated with effect design formula
   b) Justified but not satisfactory (0.5 star)
      - Justified but not estimated with effect design formula
   c) Not justified (0 star)
3) Non-respondents:
   a) Comparability between respondents and non-respondents' characteristics is established, and the response rate is satisfactory* (1 star)
      - Non-response bias measures (0.5 star)
      - ≥85% Response rate (0.5 star)
   b) The response rate is unsatisfactory, or the comparability between respondents and non-respondents is unsatisfactory (0 star)
      - No description of non-respondents or measures for non-response bias
      - <85% Response rate
   c) No description of the response rate or the characteristics of the responders and the non-responders (0 star)
4) Ascertainment of the exposure (risk factor):
   a) Validated measurement tool** (2 stars)
   b) Non-validated measurement tool, but the tool is available or described* (1 star)
   c) No description of the measurement tool (0 star)

Comparability: (Maximum 2 stars)

1) The subjects in different outcome groups are comparable, based on the study design or analysis.
   a) The study compares the outcome groups by at least two socioeconomic groups (select one outcome to compare)* (1 star)

b) The study controls the bivariate association of outcome and exposure for any additional factor (gender, body weight)* (1 star)

Outcome: (Maximum 3 stars)

1) Assessment of the outcome:
   a) Record (accelerometer)** (2 stars)
   b) Self-report diary (activities or food)** (2 stars)
   c) Self-report recall (24-recall)* (1 star)
   d) Self-report questionnaire (food-frequency questionnaire, physical activity questionnaire)* (1 star)
   e) No description
2) Statistical test:
   a) The statistical test used to analyse the data is clearly described and appropriate, and the measurement of the association is presented, including confidence intervals and the probability level (p value)* (1 star)
   b) The statistical test is not appropriate, not described or incomplete.

Table S10. NOQAS quality assessment per component of each included publications.

Author	Year data collection	Representativeness of the sample	Sample size	Non-respondents	Assessment of the exposure	Comparability	Assessment of the outcome	Statistical test	Total quality score
Dillman Carpentier et al., (2019)	2016	0,5	0,5	0	2	1	1	1	5,5
Aguilar-Farias et al., (2019)	2014 and 2015	1	0,5	0	1	1	1	1	4,5
Barranco-Ruiz et al., (2019)	2015	0	0	0	1	0	1	1	3
Berrios et al., (1990)	1986-1987	0,5	0,5	1	2	1	1	0	6
Celis-Morales et al., (2011)	2008	0	0	0	1	1	2	1	5
de Moraes Ferrari et al., (2019)	2014-2015	1	1	0	2	1	1	1	6
Ministerio de Salud de Chile, (2003)	ENS 2003	1	0,5	0,5	1	1	1	1	6
Ministerio de Salud de Chile, (2006)	2006	1	0,5	0,5	1	1	1	1	6
Ministerio de Salud de Chile, (2011)	2009-2010	1	1	1	2	2	1	1	9
Celis-Morales et al., (2016)	2009-2010	1	1	1	2	1	1	1	8
Diaz-Martínez et al., (2018)	2009-2010	1	1	1	2	1	1	1	8
Waddell et al., (2019)	2009-2010	1	0,5	0,5	2	1	1	1	6
Ministerio de Salud de Chile, (2012)	2009-2010	1	0,5	0,5	1	1	1	1	6
Ministerio de Salud de Chile, (2018)	2016-2017	1	1	0	2	1	1	0	6
Jadue et al., (1999)	1996-1997	1	1	0	2	1	1	0	6
Serón et al., (2010)	N/R	1	1	0,5	2	1	1	0	6,5

Table S11: Summary of relative differences and/or odds ratios from studies assessing associations between total physical activity and physical activity by domains and socioeconomic position indicators+.

**Study name / Author**	**Sample population**	**PA indicator**	**Education**	**Income**	**Index**
		Total physical activity			
Celis-Morales et al., (2016) / ENS 2009-10	Adults	Total PA (METs min/d)	+24.2%*	-6.6%	
ENS 2009-10	Adults	[Men] Total PA min/d	+39.6%*		
ENS 2009-10	Adults	[Women] Total PA min/d	-1.0%		
		Moderate and vigorous physical activity			
Celis-Morales et al., (2011) / GENADIO	Adults	MVPA (min/d)	+47.7%*		+8.1%
ENS 2016-17	Adults	% Vigorous PA	-OR 0.52*		
Seron et al., (2010)	Adults	% Vigorous PA	+OR 1.08		+OR 2.13
ENS 2016-17	Adults	% Moderate PA	-OR 0.95		
Seron et al., (2010)	Adults	% Moderate PA	+OR 1.53		-OR 0.88

+Relative differences (%) in physical activity between lowest and highest socioeconomic groups were estimated by: ([value highest SEP group – value lowest SEP group] / value highest SEP group) X 100. Odds ratio (OR) were reported for dichotomous outcome variables. Reference category for OR: Highest SEP group. *Relative differences >10% in physical activity, or $p<0.05$ for OR.

Differences reported are for the lowest socioeconomic group relative to the highest group (i.e.: '-' refers to lower PA engagement among the lower SEP group relative to the highest SEP group (or higher PA engagement among the highest SEP group relative to the lowest SEP group); '+' refers to the higher PA engagement among the lower SEP group relative to the lowest SEP group (or lower PA engagement among the highest SEP group relative to the lowest SEP group).

PA: Physical activity; MVPA: Moderate and vigorous physical activity; METs: Metabolic equivalents of task; min: minutes; d: day; wk: week. Moderate PA: 30min/d VPA in 3 days, or 30min/d MPA in 5 days, or ≥ 600 METs/week in 5 days; High PA: MPA≥3000 METs-min/wk in 7 days, or VPA ≥1500 METs-min/wk in 3 days.

Table S12: Summary of relative differences and/or odds ratios from studies assessing associations between transport and work-related physical activity and socioeconomic position+.

Study name / Author	Sample population	PA indicator	Education	Income	Index
		Transport-related physical activity			
Barranco-Ruiz et al., (2019)	Adults	% Active transport (walking or cycling)			-OR 0.27*
de Moraes Ferrari et al., (2019) / ELANS	Adults	Active transport (min/wk)	-26.9%*		-16.3%*
Celis-Morales et al., (2016) / ENS 2009-10	Adults	Active transport (METs min/d)	-7.2%	+1.4%	
ENS 2009-10	Adults	[Men] Active transport (min/d)	+19.9%*		
ENS 2009-10	Adults	[Women] Active transport (min/d)	-0.8%		
Waddell et al., (2019) / ENS 2009-11	Adults	% Active transport (≥600 METs min/wk)	-OR 0.59		+OR 5.58*
ENS 2016-17	Adults	% Active transport (walking or cycling)	+OR 2.58*		
Aguilar-Farias et al., (2019) / NES 2014	Adults	% Cycling for transport			+OR 1.17
Aguilar-Farias et al., (2019) / NES 2015	Adults	% Cycling for transport			+OR 1.42
		Work-related physical activity			
Celis-Morales et al., (2016) / ENS 2009-10	Adults	Work MVPA (METs min/d)	+42.5%*	-10.7%*	
ENS 2009-10	Adults	[Men] Work MVPA min/d	+78.1%*		
ENS 2009-10	Adults	[Women] Work MVPA min/d	+9.1%		

+Relative differences (%) in physical activity between lowest and highest socioeconomic groups were estimated by: ([value highest SEP group – value lowest SEP group] / value highest SEP group) X 100. Odds ratio (OR) were reported for dichotomous outcome variables. Reference category for OR: Highest SEP group. *Relative differences >10% in physical activity, or p<0.05 for OR.

Differences reported are for the lowest socioeconomic group relative to the highest group (i.e.: '-' refers to lower PA engagement among the lower SEP group relative to the highest SEP group (or higher PA engagement among the highest SEP group relative to the lowest SEP group); '+' refers to the higher PA engagement among the lower SEP group relative to the lowest SEP group (or lower PA engagement among the highest SEP group relative to the lowest SEP group).

PA: Physical activity; MVPA: Moderate and vigorous physical activity; METs: Metabolic equivalents of task; min: minutes; d: day; wk: week. Moderate PA: 30min/d VPA in 3 days, or 30min/d MPA in 5 days, or ≥ 600 METs/week in 5 days; Vigorous PA: MPA≥3000 METs-min/wk in 7 days, or VPA ≥1500 METs-min/wk in 3 days.

Table S13. Summary of relative differences and/or odds ratios from studies assessing associations between leisure time physical activity and socioeconomic position+.

Study name / Author	Sample population	PA indicator	Education	Income	Index	Occupation
		Leisure time physical activity				
Celis-Morales et al., (2016) / ENS 2009-10	Adults	Leisure time MVPA (METs min/d)	-20.6%*	+20.8%*		
ENS 2009-10	Adults	[Men] Leisure time PA (min/d)	-68.3%*			
ENS 2009-10	Adults	[Women] Leisure time PA (min/d)	-57.6%*			
de Moraes Ferrari et al., (2019) / ELANS	Adults	Leisure time PA (min/wk)	-19.6%*		-21.5%*	
ENCAVI 2006	Adults	% ≥30-min/>3 times/wk LTPA		-OR 0.31*		
ENETS 2009-2010	Adults	% ≥30-min/>3 times/wk LTPA		-OR 0.49		
ENETS 2009-2010	Adults	[Men] % ≥30-min/3 times/wk LTPA	-OR 0.22*	-OR 0.37		+OR 2.04
ENETS 2009-2010	Adults	[Women] % ≥30-min/3 times/wk LTPA	-OR 0.54	-OR 0.87		OR 1.00

+Relative differences (%) in physical activity between lowest and highest socioeconomic groups were estimated by: ([value highest SEP group – value lowest SEP group] / value highest SEP group) X 100. Odds ratio (OR) were reported for dichotomous outcome variables. Reference category for OR: Highest SEP group. *Relative differences >10% in physical activity, or p<0.05 for OR.

Differences reported are for the lowest socioeconomic group relative to the highest group (i.e.: '-' refers to lower PA engagement among the lower SEP group relative to the highest SEP group (or higher PA engagement among the highest SEP group relative to the lowest SEP group); '+' refers to the higher PA engagement among the lower SEP group relative to the lowest SEP group (or lower PA engagement among the highest SEP group relative to the lowest SEP group).

PA: Physical activity; MVPA: Moderate and vigorous physical activity; METs: Metabolic equivalents of task; min: minutes; d: day; wk: week. Moderate PA: 30min/d VPA in 3 days, or 30min/d MPA in 5 days, or ≥ 600 METs/week in 5 days; Vigorous PA: MPA≥3000 METs-min/wk in 7 days, or VPA ≥1500 METs-min/wk in 3 days.

Table S14. Summary of relative differences and/or odds ratios from studies assessing associations between physical inactivity and socioeconomic position+.

Study name / Author	Sample population	P. Inactive indicator	Education	Income	Index	Occupation
		Physical inactivity				
Berrios et al., (1990)	Adults	[Men] % <2 exercise sessions of <15 min			+OR 1.82	
Berrios et al., (1990)	Adults	[Women] % <2 exercise sessions of <15 min			+OR 2.34*	
ENS 2003	Adults	% <3 times/wk of <30-min LTPA	+OR 3.27*			
ENCAVI 2006	Adults	% <3 times/wk of <30-min LTPA	+OR 3.27*			
ENS 2009-10	Adults	% <3 times/wk of <30-min LTPA	+OR 6.76*			
ENS 2009-10	Adults	[Men] % <3 times/wk of <30-min LTPA	+OR 5.50*			
ENS 2009-10	Adults	[Women] % <3 times/wk of <30-min LTPA	+OR 6.25*			
ENS 2016-17	Adults	% <3 times/wk of <30-min LTPA	+OR 5.80*			
ENETS 2009-2010	Adults	% No LTPA/month		+OR 3.09*		
ENETS 2009-2010	Adults	[Men] % No LTPA/month	+OR 3.50*	+OR 3.65*		-OR 0.96
ENETS 2009-2010	Adults	[Women] % No LTPA/month	+OR 5.30*	+OR 1.93		OR 1.00
Jadue et al., (1999)	Adults	[Men] % No LTPA/month			+OR 1.71	
Jadue et al., (1999)	Adults	[Women] % No LTPA/month			+OR 4.62*	
		Low PA level (not meeting WHO PA guidelines)				
Celis-Morales et al., (2011) / GENADIO	Adults	Low PA level (min/d)	-1%		-7.7%	
ENS 2009-10	Adults	% Low PA level	+OR 1.25			
ENS 2009-10	Adults	% [Men] Low PA level	+OR 1.17			
ENS 2009-10	Adults	% [Women] Low PA level	+OR 1.29			
ENS 2016-17	Adults	% Low PA level	+OR 2.06*			
Serón et al., (2010)	Adults	% Low PA level	-OR 0.68		-OR 0.46	

+Relative differences (%) in physical activity between lowest and highest socioeconomic groups were estimated by: ([value highest SEP group – value lowest SEP group] / value highest SEP group) X 100. Odds ratio (OR) were reported for dichotomous outcome variables. Reference category for OR: Highest SEP group. *Relative differences >10% in physical activity, or p<0.05 for OR.

Differences reported are for the lowest socioeconomic group relative to the highest group (i.e.: '-' refers to lower PA engagement among the lower SEP group relative to the highest SEP group (or higher PA engagement among the highest SEP group relative to the lowest SEP group); '+' refers to the higher PA engagement among the lower SEP group relative to the lowest SEP group (or lower PA engagement among the highest SEP group relative to the lowest SEP group).

PA: Physical activity; MVPA: Moderate and vigorous physical activity; METs: Metabolic equivalents of task; min: minutes; d: day; wk: week. Low PA: No activity is reported, or some activity is reported but not enough for moderate or high PA levels. Moderate PA: 30min/d VPA in 3 days, or 30min/d MPA in 5 days, or ≥ 600 METs/week in 5 days; Vigorous PA: MPA≥3000 METs-min/wk in 7 days, or VPA ≥1500 METs-min/wk in 3 days.

Table S15. Summary of relative differences and/or odds ratios from studies assessing associations between sedentary behaviour (sitting time and television watching) and socioeconomic position+.

Study name / Author	Sample population	SB indicator	Education	Income	Index	Occupation
			Sitting time			
Celis-Morales et al., (2016) / ENS 2009-10	Adults	Sitting time (min/d)	-24.4%*	-23.1%*		
Celis-Morales et al., (2016) / ENS 2009-10	Adults	% >4 h of sitting time/d	-OR 0.44*	-OR 0.35*		
de Moraes Ferrari et al., (2019) / ELANS	Adults	Sitting time (min/d)	-6.7%		-14.0%*	
Diaz-Martinez et al., (2018) / ENS 2009-10	Adults	% ≥8 hours sitting time p/day	-OR 0.26*			
ENCAVI 2006	Adults	% Spends most time seated and walks little*		-OR 0.61		
ENETS 2009-2010	Adults	% Spends most time seated and walks little		-OR 0.31*		
ENETS 2009-2010	Adults	[Men] Spends most time seated and walks little	-OR 0.10*	-OR 0.32*		-OR 0.49*
ENETS 2009-2010	Adults	[Women] Spends most time seated and walks little	-OR 0.32*	-OR 0.24*		+OR 1.82
			Television watching			
Dillman Carpentier et al., (2019) / FEChiC	Children	% Used television during the week	+OR 1.56			
Dillman Carpentier et al., (2019) GOCS	Children	% Used television during the week	-OR 0.74			
Dillman Carpentier et al., (2019) / FEChiC	Children	Hours of television/wk	+14.9%*			
Dillman Carpentier et al., (2019) / GOCS	Children	Hours of television/wk	-11.1%*			

+Relative differences (%) in physical activity between lowest and highest socioeconomic groups were estimated by: ([value highest SEP group – value lowest SEP group] / value highest SEP group) X 100. Odds ratio (OR) were reported for dichotomous outcome variables. Reference category for OR: Highest SEP group. *Relative differences >10% in physical activity, or p<0.05 for OR.

Differences reported are for the lowest socioeconomic group relative to the highest group (i.e.: '-' refers to lower PA engagement among the lower SEP group relative to the highest SEP group (or higher PA engagement among the highest SEP group relative to the lowest SEP group); '+' refers to the higher PA engagement among the lower SEP group relative to the lowest SEP group (or lower PA engagement among the highest SEP group relative to the lowest SEP group).

min: minutes; wk: week; d: day; h: hour; p: per; FEChiC: Food Environment Chilean Cohort study (Mean age: 4.8 years-old); GOCS: Growth and Obesity Cohort Study (Mean age: 13.6 years-old)
